# GNAI2 polymorphic variance associates with salt sensitivity of blood pressure in the Genetic Epidemiology Network of Salt Sensitivity study

**DOI:** 10.1152/physiolgenomics.00141.2017

**Published:** 2018-06-15

**Authors:** Xiaoling Zhang, Alissa A. Frame, Jonathan S. Williams, Richard D. Wainford

**Affiliations:** ^1^Section of Biomedical Genetics, Boston University School of Medicine and The Department of Biostatistics, Boston University School of Public Health, Boston, Massachusetts; ^2^Department of Pharmacology & Experimental Therapeutics and The Whitaker Cardiovascular Institute, Boston University School of Medicine, Boston, Massachusetts; ^3^Division of Endocrinology, Diabetes, Hypertension, Brigham and Women's, Harvard Medical School, Boston, Massachusetts

**Keywords:** Gαi_2_ proteins, GNAI2, rs10510755, salt sensitivity of blood pressure

## Abstract

Salt sensitivity of blood pressure (BP) increases hypertension risk and associated adverse cardiovascular outcomes. At present, there are no validated rapid tests or diagnostic markers to identify salt sensitivity of BP in clinical practice. Based on our prior animal studies that report a role for brain Gαi_2_ proteins in the salt sensitivity of BP and evidence that GNAI2 single nucleotide polymorphisms (SNPs) associate with hypertension risk, we investigated the hypothesis that GNAI2 SNPs associate with salt sensitivity of BP in humans. Our data provide the first evidence that a GNAI2 SNP (rs10510755) positively associates with salt sensitivity of BP in the Genetic Epidemiology of Salt Sensitivity data set (continuous phenotype *P* = 0.049, case-control phenotype *P* = 0.039; *n* = 968), independently of subject sex or age. These observations suggest that genotyping at GNAI2 may be a useful biomarker in identifying individuals at risk for developing salt-sensitive BP and related complications or in identifying salt sensitivity within the hypertensive population.

## BACKGROUND/MOTIVATION FOR THE STUDY

Hypertension, the leading global noncommunicable cause of mortality (3), is projected to be the primary global cause of death and disability by 2020. Salt sensitivity of blood pressure (BP), an exaggerated pressor response to relatively higher dietary salt intake, affects ≈50% of hypertensive and ≈25% of normotensive adults (3) and increases hypertension risk (3). Salt sensitivity is an important pathotype to recognize, since it elevates risk for kidney disease and stroke independently of hypertensive effects (3). Importantly, the average U.S. salt intake (≈3.5 g/day) exceeds the American Heart Association recommendation of 1.5 g/day, making the salt sensitivity of BP a significant public health issue.

The designation of salt sensitivity of BP is typically made following an observed elevation in systolic BP (>5 mmHg) in response to sustained increases in dietary salt intake. The development of a simple diagnostic biomarker of individual salt sensitivity of BP would aid in identifying individuals at risk for developing salt sensitivity-related complications (hypertension, cardiac, renal, and cerebral diseases) and in risk stratification and treatment decisions in individuals with established salt-sensitive conditions.

Based on our prior work demonstrating a pivotal role of brain Gαi_2_ proteins in determining salt sensitivity of BP in multiple animal models (5), and data that two independent *GNAI2* SNPs in the Millennium Genome Project for Hypertension and Caucasian Italians are associated with hypertension risk (2, 4), we hypothesized that *GNAI2* genetic variation would be associated with the salt sensitivity of BP in the Genetic Epidemiology of Salt Sensitivity (GenSalt) data set (1).

## PHENOTYPE

In this analysis, salt sensitivity of BP was defined as the change in systolic BP observed between a 7-day restricted-sodium feeding (51.3 mmol/day) and a 7-day high-sodium feeding (307.8 mmol/day). As per GenSalt program design as previously reported (1), all BP readings were measured in the morning by trained and certified observers using a random-zero sphygmomanometer after 5 min of rest with the participant in the sitting position and the arm placed at the level of the heart. The BP levels during the dietary sodium intervention were calculated as the mean of nine measurements obtained from three clinical visits on *days 5, 6*, and *7* of each dietary sodium intervention phase (1). Salt sensitivity of BP was defined by the observation of a 5 mmHg (4.6–4.9 mmHg rounded to 5 mmHg) or greater increase in mean systolic BP following the transition from a restricted to high-sodium intake (i.e., BP response to high-sodium diet = BP on high-sodium diet minus BP on restricted-sodium diet).

### 

#### Cohort details.

The GenSalt population cohort study has been extensively defined previously. In brief, the GenSalt study was conducted in a Han Chinese population in rural north China during 2003-05. The study was approved by a Human Research Oversight committee, and written consent was obtained from each participant. This population had a mean systolic BP that ranged from 130 to 160 mmHg without use of antihypertensive medications. All subjects were between 18 and 60 yr, with a mean age of 38.5 ± 6 yr (1). A total of 1,000 subjects in this study underwent whole genome genotyping using the Affymetrix Genome Wide Human SNP Array AFFY_6.0. Of this population, 968 had complete genotype and phenotype data available for analysis. Among these 968 subjects, we categorized 369 as “salt sensitive” (175 females, 194 males) and 599 as “nonsalt sensitive” (330 females, 269 males). For the salt sensitive subjects, the mean ± SD of BP response to high-sodium diet is 7.82 ± 3.20. For the nonsalt-sensitive subjects, the mean ± SD of BP response to high-sodium diet is −0.05 ± 3.16.

#### Type of study.

Candidate gene.

#### Details of the single nucleotide polymorphisms studied.

The candidate gene under investigation was GNAI2. Primary single nucleotide polymorphisms (SNPs) investigated were rs10510755, rs9852677, rs2282751, rs4547694, and rs2298952 identified from HapMap and 1000Genomes projects to capture 100% of the genetic variation in GNAI2.

#### Analysis model.

For the association analysis, the continuous phenotype was the difference in mean systolic BP from a low- to a high-salt intake, which represents the quantitative trait of the salt sensitivity of BP (i.e., BP response to high sodium diet = BP on high-sodium diet minus BP on restricted-sodium diet). Linear regression was used to model salt sensitivity of BP and an additive genetic model (SNP dosage) adjusted for age, sex, and principal components. In addition, we conducted a logistic regression analysis, which provides an odds ratio (OR) estimate, by treating the binary trait (salt sensitive vs. nonsalt sensitive) as the outcome; SNP as predictor; and age, sex, and principal components as covariates. For all analyses, the significance threshold was considered at *P* < 0.05.

## RESULTS

Of the five GNAI2 SNPs selected for testing, only SNPs rs10510755, rs9852677, and rs2282751 were present in GenSalt after quality control. As such, only these three SNPs underwent analysis. SNPs rs9852677 and rs2282751 were not significantly associated with the salt sensitivity of BP in the GenSalt data set (Supplemental Table S1). (The online version of this article contains supplemental material.) However, rs10510755 was positively associated with salt sensitivity of BP (continuous phenotype *P* = 0.049, case-control phenotype *P* = 0.039; Supplemental Table S1). For SNP rs10510755, the minor allele frequency (MAF) was 6.2% in the GenSalt population. The GenSalt data set contains 369 salt-sensitive individuals in which SNP rs10510755 was identified in 118 independent subjects. The minor allele at rs10510755 was more frequently observed within the salt-sensitive cohort compared with the nonsalt-sensitive cohort (salt-sensitive cohort 32% vs. nonsalt-sensitive cohort 12%, *P* < 0.05) even after adjusting for age and sex. There was no additive SNP effect observed, however; only one subject exhibited two minor alleles in this study. SNP rs10510755 is associated with greater odds of the salt sensitivity of BP (case-control phenotype OR 3.046; 95% confidence intervals 2.195–4.228, Z-score 6.663, *P* = 0.039). This suggests that subjects with GNAI2 SNP rs10510755 are three times more likely to be salt sensitive than subjects that lack SNP rs10510755.

## INTERPRETATION

These results suggest a positive association between the GNAI2 SNP rs10510755 and salt sensitivity of BP and provide important clinical relevance to our prior mechanistic work in animal models regarding the influence of Gαi_2_ proteins on the salt sensitivity of BP (5). Possible limitations of the current analysis are the relatively small sample size, the lack of ability to include analysis of body mass index as a covariate, and the low MAF of the indexed SNP. These data suggest GNAI2 polymorphic variance represents a potential biomarker for salt sensitivity of BP that may identify a specific subset (≈32% based on GenSalt data set) of salt-sensitive subjects.

## GRANTS

National Institutes of Health (NIH) Grants R01HL-107330, K02HL-112718, R01HL-141406, R01HL-139867, 1UL1TR-001430; American Heart Association Grants AHA17GRNT33670023, AHA16MM32090001 to R. D. Wainford; NIH Grants F31DK-116501 to A. A. Frame, NIH U01AG-032984, UF1AG-046198, R01AG-048927 to X. Zhang.

## DISCLOSURES

No conflicts of interest, financial or otherwise, are declared by the authors.

## AUTHOR CONTRIBUTIONS

X.Z., J.S.W., and R.D.W. performed experiments; X.Z., A.A.F., J.S.W., and R.D.W. analyzed data; X.Z., A.A.F., J.S.W., and R.D.W. interpreted results of experiments; X.Z. and R.D.W. prepared figures; X.Z., A.A.F., J.S.W., and R.D.W. edited and revised manuscript; X.Z., A.A.F., J.S.W., and R.D.W. approved final version of manuscript; A.A.F., J.S.W., and R.D.W. conceived and designed research; A.A.F., J.S.W., and R.D.W. drafted manuscript.

## Supplemental Data

Table 1 Table 1 (.xlsx 13 KB)

Table 2 Table 2 (.xlsx 14 KB)
